# The impact of androgen receptor pathway inhibitors as starting treatment in metastatic castration-sensitive prostate cancer on patient outcomes (OASIS Japan)

**DOI:** 10.1038/s41598-025-93136-9

**Published:** 2025-04-19

**Authors:** Masaki Shiota, Yanfang Liu, Suneel Mundle, Mehregan Nematian-Samani, Jason Hwang, Xiayi Wang, Hirotsugu Uemura

**Affiliations:** 1 Department of Urology, Graduate School of Medical Sciences, Kyushu University, Fukuoka, Japan; 2 Janssen Pharmaceuticals LLC, Raritan, USA; 3 Janssen Research & Development, Raritan, USA; 4 Janssen-Cilag GmbH, Neuss, Germany; 5 Janssen Pharmaceutical Kabushiki Kaisha, Tokyo, Japan; 6 Janssen Global Services, LLC, Titusville, NJ USA; 7 Department of Urology, Kindai University Faculty of Medicine, Osaka-Sayama, Japan

**Keywords:** Androgen receptor signalling inhibitors, Apalutamide, Metastatic castration-sensitive prostate cancer, Overall survival, Prostate cancer, Outcomes research

## Abstract

We examined the impact of starting treatment on clinical outcomes in men with metastatic castration-sensitive prostate cancer (mCSPC). This retrospective observational cohort study used claims data from the Medical Data Vision (MDV) hospital-based administrative dataset in Japan. All patients with newly diagnosed mCSPC from 1 January 2018 to 31 March 2024 were enrolled and followed up until 30 September 2024. Time-to-event analyses used Kaplan-Meier methods. The risk of death, onset of castration resistance, time to ≥ 50% PSA decline (PSA50), ≥ 90% PSA decline (PSA90), and undetectable PSA level (≤ 0.2 ng/mL) was compared between androgen receptor pathway inhibitors (ARPIs) and combined androgen blockade (CAB) or androgen-deprivation therapy (ADT) alone using a Cox proportional hazard model adjusted for age, body mass index, co-morbidities, visceral metastases, and baseline PSA. 22,559 patients with mCSPC had received relevant treatment of whom 15,797 were included in the analysis: 1167 (5.2%) started on apalutamide (APA) + ADT, 1407 (6.2%) on enzalutamide + ADT, 1262 (5.6%) on abiraterone acetate plus prednisone + ADT, and 11,961 (53.0%) on CAB/ADT alone. The median age was between 74 and 78 years in each group. Bone metastases were present in 60.5% to 72.6% of patients, visceral metastases in 2.8% to 5.7%, and nodal metastases in 19.3% to 29.4%. Overall survival and castration resistance-free survival were significantly longer in patients initially treated with APA + ADT compared to CAB/ADT (*p* < 0.0001 for both comparisons). In patients with regular PSA assessment, a higher percentage of patients starting with APA + ADT achieved PSA50, PSA90 and undetectable PSA at 3 months compared with CAB/ADT (*p* < 0.0001, *p* = 0.0005, *p* < 0.0001, respectively). Use of APA + ADT as a starting treatment for mCSPC was associated with better clinical outcomes versus traditional CAB or ADT in real-world clinical practice in Japan.

**Supplementary Information:**

The online version contains supplementary material available at 10.1038/s41598-025-93136-9.

## Introduction

Prostate cancer (PC) is the fourth most common cancer in men in Asia and with 370,000 new cases in 2020^1^. Japan has the highest incidence rate of PC in Asia, with an age-standardized rate of 51.8 per 100,000 population. In 2020, there were around 106,000 new cases and more than 13,000 deaths due to PC in Japan^1^. Metastatic disease at diagnosis is common in the absence of population-based screening programmes in most of Asia, and in Japan, around 20% of patients presenting with PC have *de novo* metastases^2^. The 5-year survival for metastatic castration-sensitive PC (mCSPC) is around 30% globally^3^. In Japan, 5-year of survival of patients with stage IV mCSPC is 51%^4^.

Androgen-deprivation therapy (ADT) was the standard of care for mCSPC for many decades^5^. The availability of androgen receptor pathway inhibitors (ARPIs – apalutamide [APA], enzalutamide [ENZ], darolutamide [DARO], abiraterone acetate plus prednisone [AAP]) has promoted rapid evolution of the treatment landscape and improved patient outcomes^6^. Clinical trials have demonstrated clinically and statically significant survival benefits using upfront dual combination therapy with AAP + ADT (LATITUDE trial), ENZ + ADT (ENZAMET and ARCHES trials), and APA + ADT (TITAN trial) in some patient groups^7–11^. Upfront ARPI combination therapy has become the standard of care, replacing ADT and combined antigen blockade (CAB) alone^12,13^. More recent studies (PEACE-1 and ARASENS trials) suggest that further clinical benefits can be achieved using triple combination therapy with an ARPI + ADT + docetaxel (DTX)^14,15^.

APA and ENZ are oral ARPIs that bind to the androgen receptor, preventing androgen receptor-mediated transcription and inhibiting PC cell proliferation. The TITAN trial was a Phase 3, randomized, placebo-controlled study that showed significantly improved overall survival (OS) and radiographic progression-free survival (rPFS) in patients with mCSPC treated with APA + ADT compared to ADT alone. Combined therapy significantly delayed the onset of castration resistance, and reduced the risk of death by 35% (*p* < 0.0001) compared to ADT alone^9,10^. A rapid and deep PSA decline to ≤ 0.2 ng/mL (‘undetectable’) and a 90% decrease from baseline (PSA90) in the first 3 months of treatment were associated with longer OS, longer time to PSA progression, and longer time to castration resistance, compared with patients who did not achieve a decline in PSA (*P* < 0.0001 for all comparisons)^16^.

In the ARCHES trial, ENZ + ADT significantly reduced the risk of disease progression and reduced the risk of death by 34% (*p* < 0.001) compared to ADT alone in men with mCSPC. During the study, 68.1% of patients treated with ENZ + ADT reached undetectable PSA versus 17.6% in the ADT alone group, and time to castration resistance was significantly prolonged (*p* < 0.001 for both comparisons)^11^. In the ENZEMET trial, 67% of patients treated with ENZ + ADT were alive at 5 years versus 57% treated with standard-of-care + ADT^17^. Apalutamide and ENZ were approved for the treatment of patients with mCSPC in Japan in 2020^18,19^.

Abiraterone is a selective inhibitor of CYP17A1 which is pivotal for androgen synthesis. In the LATITUDE trial, AAP + ADT significantly improved rPFS and reduced the risk of death by 38% (*p* < 0.001) compared with ADT alone in patients with mCSPC^7^. A 50% reduction in PSA from baseline (PSA50) was achieved by 91% of patients in the AAP + ADT group versus 67% in the ADT alone group (*p* < 0.001). Abiraterone plus prednisone was approved for the treatment of patients with mCSPC in Japan in 2018^20^.

The array of treatment choices for mCSPC allows physicians to personalise patient management; however, data are lacking to guide selection of the starting treatment to achieve optimal clinical outcomes. We conducted a retrospective, observational cohort study to examine the impact of the starting treatment on PSA outcomes, onset of castration resistance, and OS in patients with mCSPC treated in real-world clinical practice in Japan.

## Patients and methods

### Data source

Medical Data Vision (MDV) is a longitudinal nationwide hospital-based claims database that holds data for more than 38 million patients treated at approximately 300 hospitals in Japan (around 20% of total number of hospitals) that participate in the diagnosis procedure combination/per-diem payment system^20^. The database contains anonymized information about diagnoses (coded using International Classification of Disease, version 10 [ICD-10]), patient characteristics, drugs prescribed in hospital or outpatient clinics, medical procedures, laboratory test results, features of medical facilities, and reimbursement costs.

### Ethical approval and consent to participate

The database containing anonymized data was licensed from MDV to Janssen Pharmaceutical Kabushiki Kaisha. Patient information was linked via anonymized patient identifiers assigned in the database. Based on the Ethical Guidelines on Biomedical Research Involving Human Subjects (Ministry of Education, Culture, Sports, Science and Technology, and Ministry of Health, Labour and Welfare of Japan), individual informed consent and ethical approval were not applicable to this study. The study was approved by the sponsor’s internal Medical Review Board committee and was performed in accordance with relevant guidelines and regulations.

### Study design and population

The study population comprised all men at least 18 years of age with a diagnosis of mCSPC during the study period from 01 January 2018 until 31 March 2024. Patients were required to have at least 2 claims for a diagnosis of PC (ICD-10 code C61), and at least 1 claim with a diagnosis of metastatic disease on, or after, the first observed PC diagnosis during the study period, with no prior claim for metastatic disease, and no claim with a diagnosis of any other type of cancer (other than non-melanoma skin cancer or bladder cancer treated with transurethral resection of the tumour only) prior to the index date, using all available data from the beginning of data stream. Patients were to meet at least one of the following criteria to confirm hormone sensitivity:

- Castration/castration naïve, defined as no claims/ diagnosis of castration resistance prior to the index date (using all available data from the beginning of data stream) and no claims for ADT in the 18 months prior to the index date,
- At least one claim for surgical castration and at least two PSA test results (including one nadir and one post-nadir) after the surgical castration and within 12 months prior to or on the index date without evidence of biochemical progression (PSA level ≥ 2 ng/mL and ≥ 25% increase from nadir),
- Medical castration (at least 90 days of continuous ADT) prior to the index date and at least 2 PSA test results (including one nadir and one post-nadir) within the same continuous ADT episode and within 12 months prior to or on the index date, without evidence of biochemical progression (PSA level ≥ 2 ng/mL and ≥ 25% increase from nadir).

The study cohort included all eligible patients who started treatment with either: APA + ADT, ENZ + ADT, AAP + ADT, DARO + DTX + ADT, DTX + ADT, an ADT regimen alone, CAB, surgical castration (bilateral orchiectomy), or radiation therapy. The index date was the date of the first ARPI treatment (CAB/ADT alone if no ARPI was prescribed) received after a metastatic diagnosis during the study period. Patients were followed up for at least 6 months or until death, loss to follow-up, or the end of the study follow-up period (30 September 2024), whichever occurred first.

### Outcomes

Clinical outcomes were OS, castration resistance-free survival, time to a ≥ 50% decline (PSA50) and ≥ 90% decline (PSA90) in PSA from the latest baseline value, and time to undetectable PSA level (≤ 0.2 ng/mL). Patients without an event, or patients with no record of a medical visit for at least 12 months prior to study end (lost to follow-up) were censored at the last follow-up visit. OS, castration resistance-free survival, and time to PSA50, PSA90 and undetectable PSA were calculated from the index date to the date of the event. Castration resistance during the follow-up period was identified by a claim for castration resistance, a change in treatment, and a record of rising PSA if available.

Clinical outcomes were compared between groups initiated on an ARPI + ADT and patients initiated on CAB/ADT.

### Statistical analysis

Baseline demographic and clinical characteristics, when available in the database, were summarized using descriptive statistics. Continuous variables were summarized by median, and quartiles (Q1, Q3). Frequencies and percentages were provided for categorical variables. For patients with PSA results, PSA-related outcomes were summarized using descriptive statistics for all patients with sufficient data and then stratified by treatment. Time-to-event variables were summarized by the median survival time with 95% confidence intervals (CIs) and survival probabilities with 95% CIs based on the Kaplan-Meier method, and their differences were examined by log-rank test. Relative risks of onset of castration resistance, death, and achieving PSA50, PSA90 and undetectable PSA were estimated using multivariate Cox proportional hazard models adjusted for age, Charlson Co-morbidity Index score, body mass index, presence of visceral metastases, and baseline PSA. All tests were two-sided, and *p* < 0.05 was considered statistically significant.

## Results

### Study cohort

**Fig. 1:**
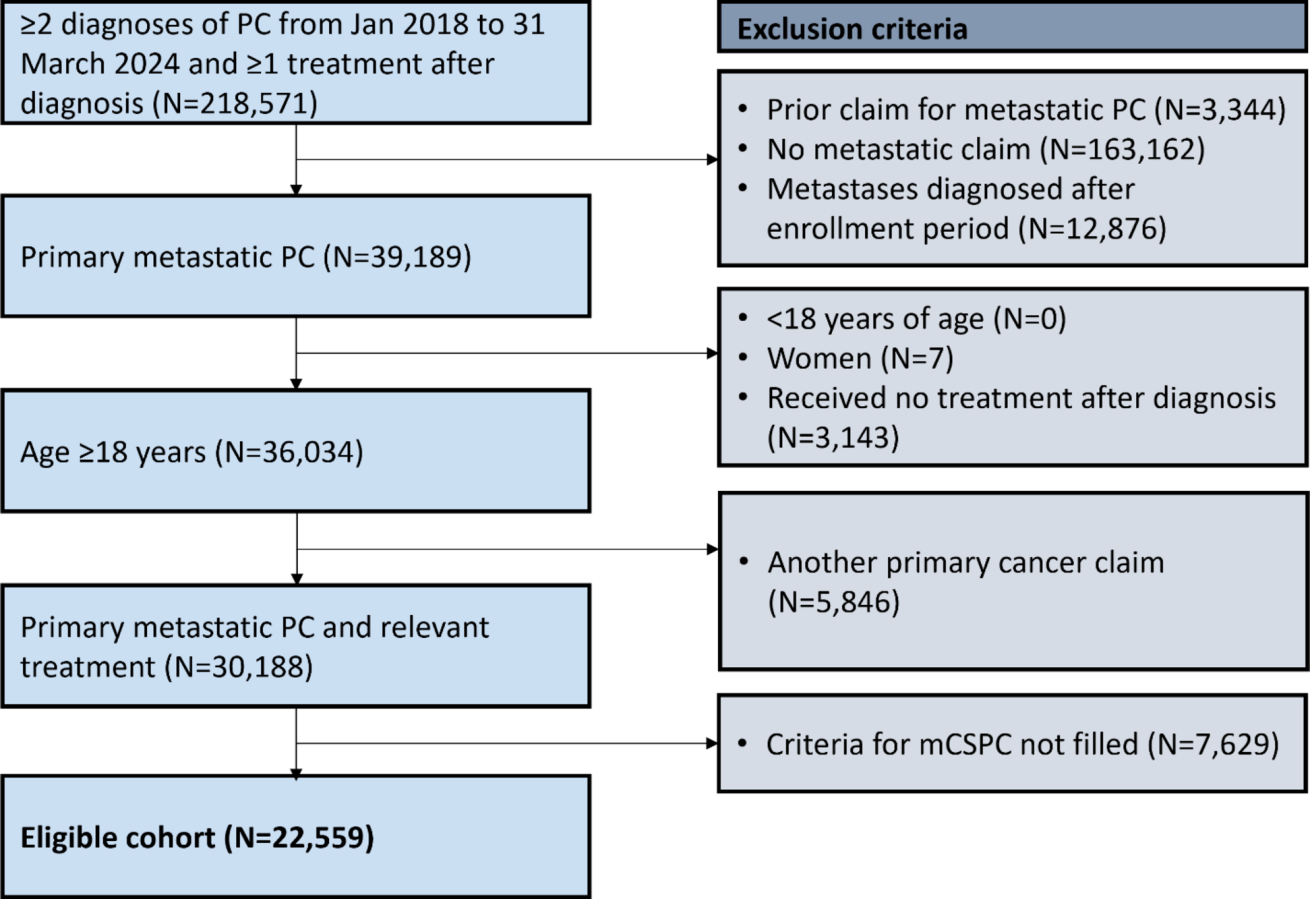
Patient selection flowchart. PC, prostate cancer; mCSPC, metastatic castration-sensitive prostate cancer; N, number of patients.

There were 22,559 eligible patients with a new diagnosis of mCSPC who received a relevant treatment between January 2018 and 31 March 2024 (Fig. 1). Of these patients, 1167 received upfront treatment with APA + ADT, 1407 received ENZ + ADT, 1262 received AAP + ADT, and 11,961 received CAB/ADT alone (Table 1). There were 314 additional patients who received DTX + ADT, 2407 who received radiotherapy, and 4041 who were ineligible for analysis due to the use of other treatments or follow-up less than 6 months. Given the clinical heterogeneity of these groups, particularly those treated with first line DTX + ADT, radiotherapy, and other treatments, we restricted our comparative analysis to patients who received ARPIs compared with CAB/ADT. Additionally, there were 166 patients treated with DARO + DTX + ADT triple therapy; however the follow-up time was limited and these patients were not included further in the study.

**Table 1:** Demographic and clinical characteristics of patients at baseline, by starting treatment.

	APA + ADT *N* = 1167	ENZ + ADT *N* = 1407	AAP + ADT *N* = 1262	CAB/ADT *N* = 11,961
**Age**, median (Q1, Q3)	74 (69, 79)	76 (71, 81)	74 (69, 79)	78 (72, 84)
**BMI**, median (Q1, Q3)	23.0 (21.1, 25.1)	23.0 (20.7, 25.1)	22.4 (20.3, 24.5)	22.5 (20.3, 24.7)
**Site of metastases**, n (%)				
Bone	720 (61.7)	922 (65.5)	916 (72.6)	7242 (60.5)
Visceral	40 (3.4)	52 (3.7)	35 (2.8)	678 (5.7)
Nodal	343 (29.4)	379 (26.9)	244 (19.3)	3035 (25.4)
Baseline PSA*, median (Q1, Q3)	9.2 (1.0, 144.3)	8.9 (1.0, 94.0)	10.5 (0.9, 156.3)	13.9 (0.9, 229.7)
**CCI**, median (Q1, Q3)	0.0 (0.0, 1.0)	0.0 (0.0, 1.0)	0.0 (0.0, 1.0)	1.0 (0.0, 1.0)
0	683 (58.5)	775 (55.1)	640 (50.7)	6420 (53.7)
1	280 (24.0)	347 (24.7)	376 (29.8)	2808 (23.5)
2	108 (9.3)	146 (10.4)	147 (11.6)	1365 (11.4)
3	44 (3.8)	65 (4.6)	59 (4.7)	708 (5.9)
4	27 (2.3)	43 (3.1)	26 (2.1)	367 (3.1)
5 +	25 (2.1)	31 (2.2)	14 (1.1)	293 (2.4)
**Comorbiditie**s, n (%)				
Acute myocardial infarction	14 (1.2)	11 (0.8)	7 (0.6)	144 (1.2)
Cerebrovascular disease	61 (5.2)	107 (7.6)	84 (6.7)	1193 (10.0)
Chronic obstructive pulmonary disease	37 (3.2)	65 (4.6)	59 (4.7)	682 (5.7)
Congestive heart failure	113 (9.7)	162 (11.5)	114 (9.0)	1553 (13.0)
Dementia	9 (0.8)	17 (1.2)	8 (0.6)	278 (2.3)
Diabetes	168 (14.4)	172 (12.2)	120 (9.5)	1338 (11.2)
Diabetes with complications	30 (2.6)	40 (2.8)	17 (1.3)	320 (2.7)
History of myocardial infarction	24 (2.1)	29 (2.1)	22 (1.7)	277 (2.3)
Mild liver disease	112 (9.6)	152 (10.8)	194 (15.4)	1231 (10.3)
Moderate/severe liver disease	2 (0.2)	9 (0.6)	2 (0.2)	37 (0.3)
Paralysis	5 (0.4)	7 (0.5)	12 (1.0)	95 (0.8)
Peptic ulcer disease	110 (9.4)	158 (11.2)	232 (18.4)	1413 (11.8)
Peripheral vascular disease	18 (1.5)	35 (2.5)	28 (2.2)	284 (2.4)
Renal disease	65 (5.6)	75 (5.3)	51 (4.0)	763 (6.4)
Rheumatologic Disease	7 (0.6)	6 (0.4)	7 (0.6)	54 (0.5)
**Duration of treatment (m)**, median (Q1, Q3)	9.1 (3.7, 19.3)	10.1 (3.9, 19.3)	12.9 (4.0, 26.0)	9.1 (3.5, 20.7)
**Duration of follow-up (m)**, median (Q1, Q3)	17.9 (10.3, 29.4)	14.2 (7.8, 23.4)	22.0 (11.7, 36.5)	20.5 (9.1, 39.1)

AAP, abiraterone acetate plus prednisone; ADT, androgen deprivation therapy; APA, apalutamide; BMI, body mass index; CAB, combined androgen blockade; CCI, Charlson Comorbidity Index; ENZ, enzalutamide; m, months; Q1, Q3, first and third quartiles.

*Within 30 days before first ARPI treatment commencement (CAB/ ADT alone if no ARPI was prescribed); the latest PSA measurement was used if multiple PSA measurements were available within the 30 days; APA + ADT (*n* = 179), ENZ + ADT (*n* = 202), AAP + ADT (*n* = 182), and CAB/ADT (*n* = 1758).

The median age of patients at the index date was between 74 years (AAP + ADT group) and 78 years (CAB/ADT group) in each group. Bone metastases were present in 61.7% of patients in the APA + ADT group, 65.5% in the ENZ + ADT group, 72.6% in the AAP + ADT groups, and 60.5% in the CAB/ADT group. Visceral metastases were present in 2.8% to 5.7% of patients, and nodal metastases in 19.3% to 29.4%. The most frequently occurring comorbidities were peptic ulcer disease (9.4% to 18.4% of patients across treatment groups), diabetes (11.2% to 14.4%), mild liver disease (9.6% to 15.4%), and congestive heart failure (9.0% to 13.0%).

The median duration of starting treatment ranged from 9.1 to 12.9 months. The median follow-up period was 14.2 to 22.0 months across groups (Table 1).

### Castration resistance-free survival

**Fig. 2:**
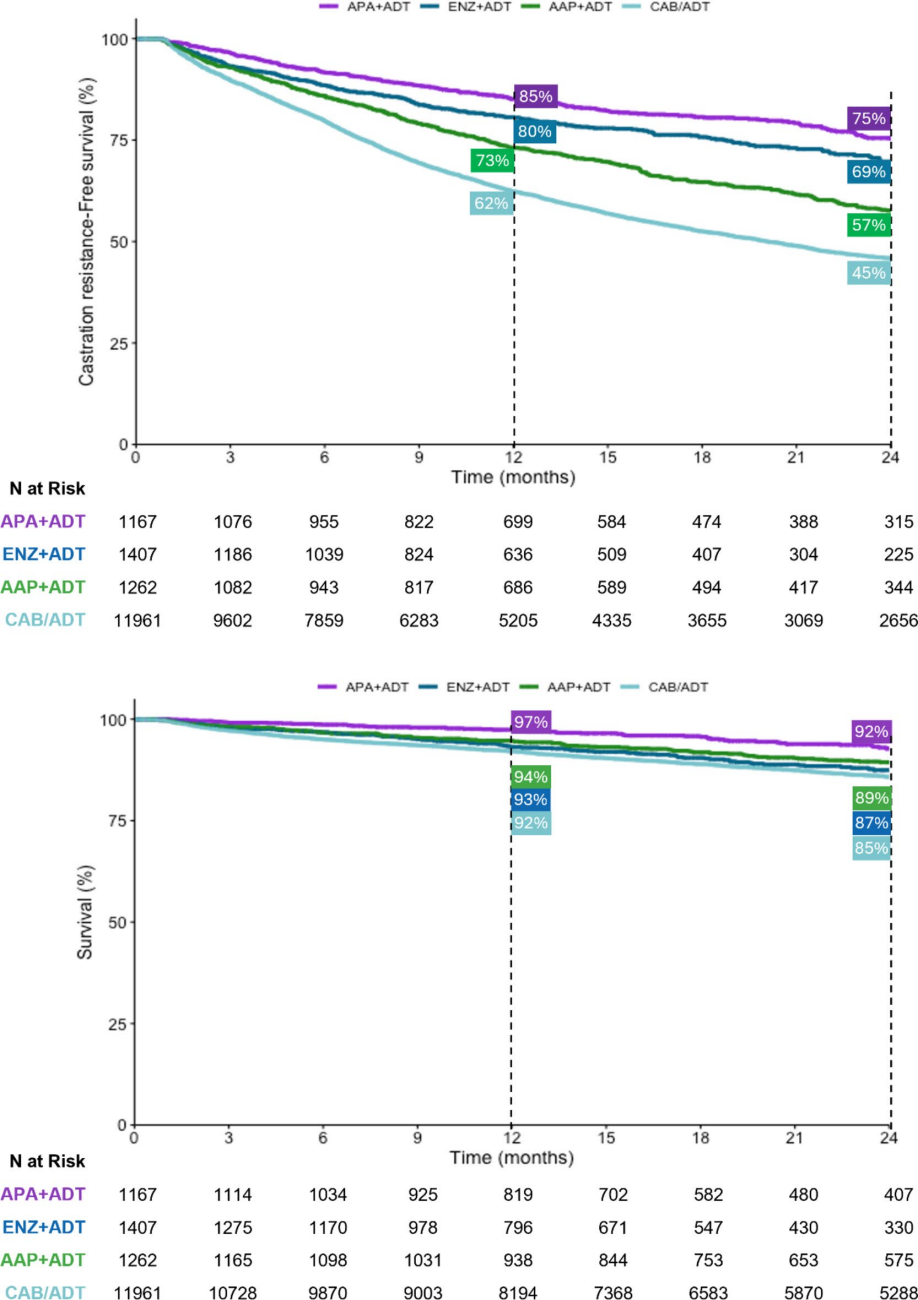
Castration resistance-free survival and overall survival in patients with metastatic castration-sensitive prostate cancer, by starting treatment. AAP, abiraterone acetate plus prednisone; ADT, androgen deprivation therapy; APA, apalutamide; CAB, combined androgen blockade; ENZ, enzalutamide.

After 12 months, 85% of patients who started treatment for mCSPC with APA + ADT remained castration sensitive, versus 80% who started treatment with ENZ + ADT, 73% with AAP + ADT, and 62% with CAB/ADT (Fig. 2). At 24 months the percentage was 75% in the APA + ADT group, versus 69%, 57%, and 45% in the respective groups.

**Fig. 3:**
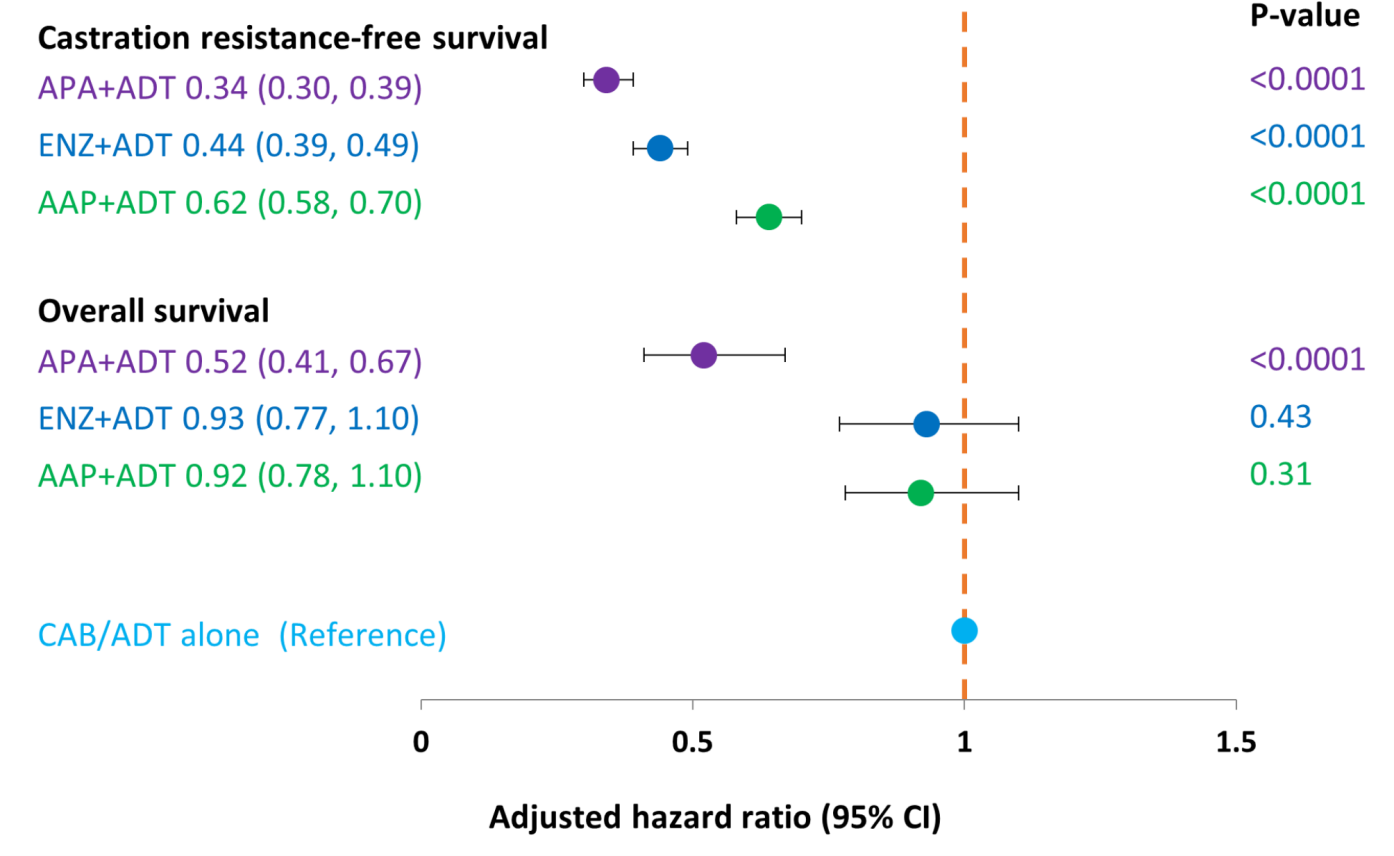
Multivariate Cox regression model results for hazard ratio of long-term outcomes, by starting treatment in metastatic castration-sensitive prostate cancer as compared to CAB/ADT alone (Kaplan-Meier method). AAP, abiraterone acetate plus prednisone; ADT, androgen deprivation therapy; APA, apalutamide; CAB, combined androgen blockade; CI, confidence interval; ENZ, enzalutamide. p-value = log rank test comparing each ARPI + ADT group versus CAB/ADT group.

In the multivariate Cox regression model adjusted for age, body mass index, comorbidities, visceral metastases, and PSA at baseline, castration resistance-free survival was significantly longer in the APA + ADT, ENZ + ADT and AAP + ADT groups compared to CAB/ADT (*p* < 0.0001 for all comparisons) (Fig. 3).

### Overall survival

OS at 24 months was 92% in patients started treatment with APA + ADT versus 89% in the ENZ + ADT group, 87% in the AAP + ADT group, and 85% in the CAB/ADT group (Fig. 2). Median OS was not reached in any group by 24 months. Similar results were observed when CAB and ADT alone were assessed separately, with the lowest 24-month survival observed for ADT alone (**Fig.** **S1**).

In the adjusted multivariate Cox regression model, OS was significantly longer in the APA + ADT group compared to the CAB/ADT group (*p* < 0.0001) and was not significantly different for ENZ + ADT and AAP + ADT compared to CAB/ADT (Fig. 3).

### PSA responses

#### Time to PSA50 and PSA90

**Fig. 4:**
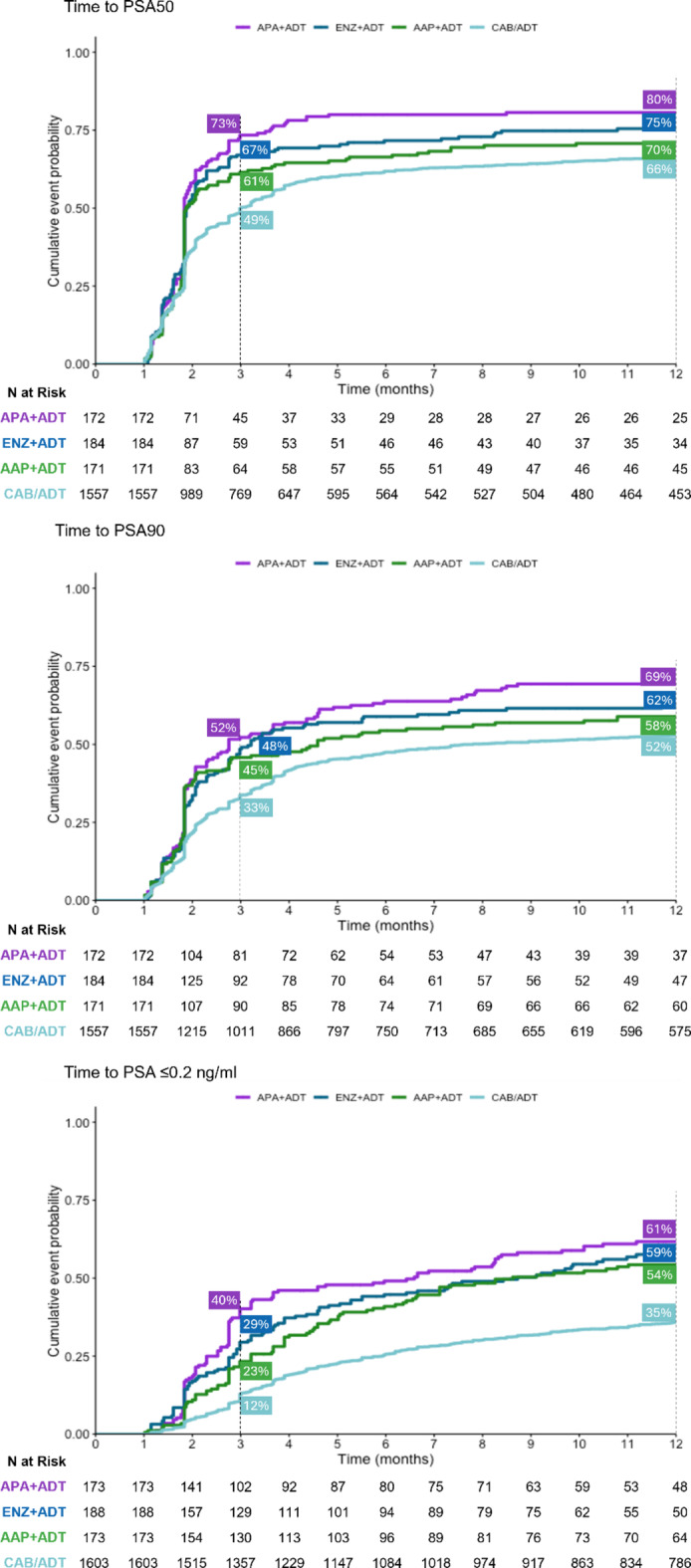
PSA responses in patients with metastatic castration-sensitive prostate cancer, by starting treatment. AAP, abiraterone acetate plus prednisone; ADT, androgen deprivation therapy; APA, apalutamide; CAB, combined androgen blockade; ENZ, enzalutamide.

After 3 months, 73% of patients who started treatment for mCSPC with APA + ADT had reached PSA50, versus 67% who started treatment with ENZ + ADT, 61% with AAP + ADT, and 49% with CAB/ADT (Fig. 4). By 12 months, 80% of patients in the APA + ADT group had reached PSA50, versus 75% in the ENZ + ADT group, 70% in the AAP + ADT group, and 66% in the CAB/ADT group.

**Fig. 5:**
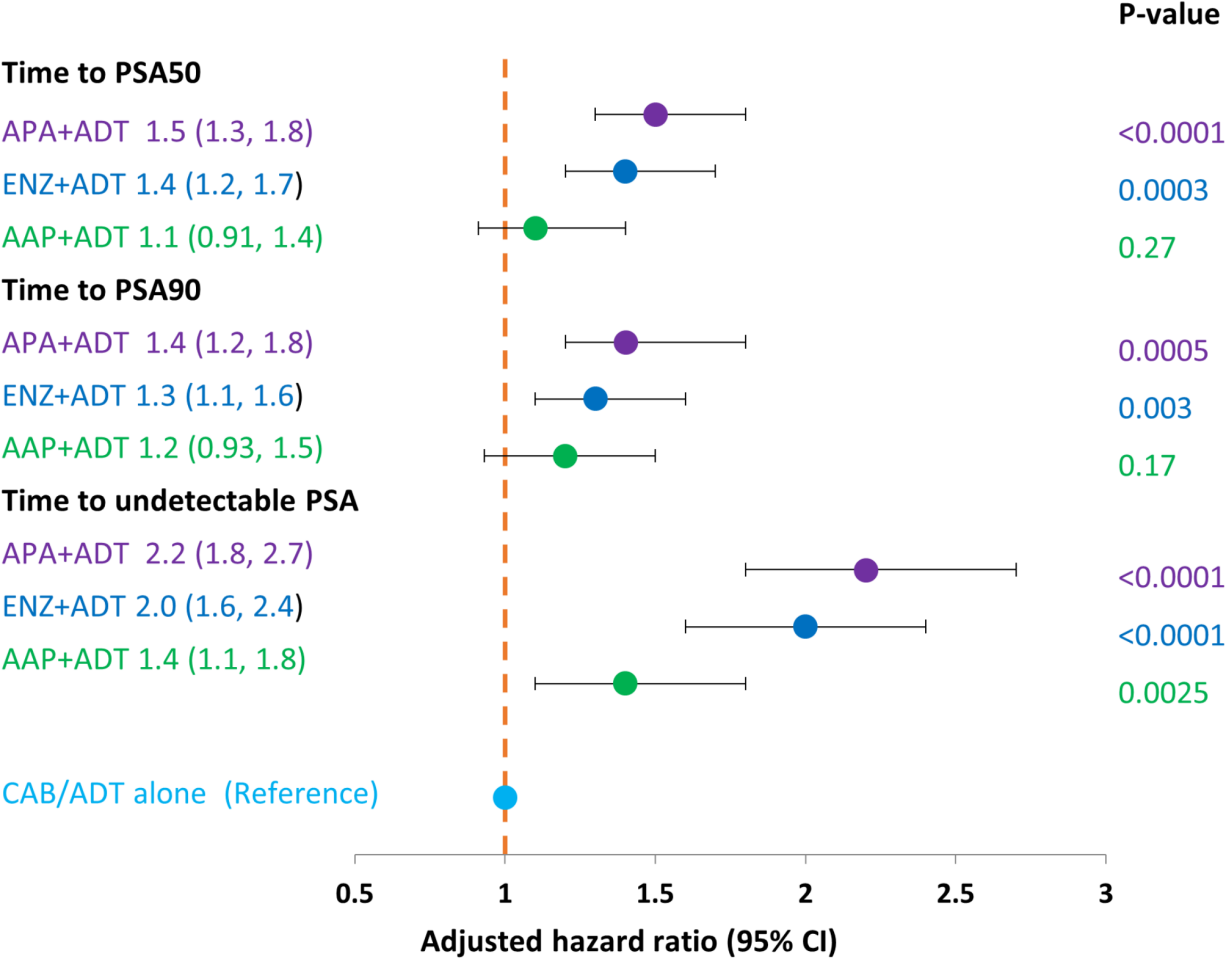
Multivariate Cox regression model results for hazard ratio of short-term outcomes, by starting treatment in metastatic castration-sensitive prostate cancer as compared to CAB/ADT alone (Kaplan-Meier method). AAP, abiraterone acetate plus prednisone; ADT, androgen deprivation therapy; APA, apalutamide; CAB, combined androgen blockade; CI, confidence interval; ENZ, enzalutamide; PSA50, time to ≥ 50% PSA decline from baseline; PSA90), time to ≥ 90% PSA decline from baseline; Undetectable PSA, level ≤ 0.2 ng/mL. p-value = log rank test comparing each ARPI + ADT group versus CAB/ADT group.

In the multivariate Cox regression model, the likelihood of reaching PSA50 was significantly higher in the APA + ADT and ENZ + ADT groups compared to CAB/ADT (*p* < 0.0001 and *p* = 0.0003, respectively) and was not significantly different for AAP + ADT compared to CAB/ADT (Fig. 5).

#### Time to PSA90

PSA90 was reached at 3 months by 52% of patients who started treatment for mCSPC with APA + ADT, versus 48% who started treatment with ENZ + ADT, 45% with AAP + ADT, and 33% with CAB/ADT (Fig. 4). By 12 months, 69% of patients in the APA + ADT group had reached PSA90 versus 62% in the ENZ + ADT group, 58% in the AAP + ADT group, and 52% in the CAB/ADT group.

In the adjusted multivariate Cox regression model, the likelihood of reaching PSA90 was significantly higher in the APA + ADT and ENZ + ADT groups compared to CAB/ADT (*p* = 0.0005, and *p* = 0.003, respectively), but was not significant for the AAP + ADT group compared to CAB/ADT (Fig. 5).

#### Time to undetectable PSA (≤ 0.2 ng/ml)

At 3 months, 40% of patients who started treatment for mCSPC with APA + ADT had undetectable PSA, versus 29% who started treatment with ENZ + ADT, 23% with AAP + ADT, and 12% with CAB/ADT (Fig. 4). By 12 months, 61% of patients in the APA + ADT group had undetectable PSA versus 59% in the ENZ + ADT group, 54% in the AAP + ADT group, and 35% in the CAB/ADT group. Similar results were observed when CAB and ADT alone were assessed separately (**Fig.** **S1**).

In the adjusted multivariate Cox regression model, the likelihood of achieving undetectable PSA was significantly higher in the APA + ADT, ENZ + ADT and AAP + ADT groups compared to CAB/ADT (*p* ≤ 0.0025 for all comparisons) (Fig. 3).

## Discussion

This study confirmed that in real world clinical practice, ARPIs provided clinical benefits to patients when used as starting treatment for mCSPC. APA + ADT, ENZ + ADT, and AAP + ADT all showed statistically significant benefits over CAB/ADT alone in prolonging the time until development of castration resistance and increasing the likelihood of reaching undetectable PSA. APA + ADT was the only treatment to have a statistically significant impact on improving survival, and the only treatment that induced statistically significant improvements in all of the outcomes evaluated compared to CAB/ADT alone. Therefore, it may be reasonable to hypothesize that APA + ADT may provide a clinical benefit over the other life-prolonging therapies assessed in this study. This hypothesis is supported by other reports in the literature; real-world studies in the United States found that patients with mCSPC started on APA were more likely to reach PSA90 and achieved this response earlier than patients started on AAP or ENZ^21,22^. In these studies, patients who started APA were 53% more likely to reach PSA90 at 6 months than those initiated on AAP, and 56% more likely than those started on ENZ^21,22^.

Early and deep PSA responses are associated with improved prognosis in metastatic PC^23^. A *post hoc* analysis of the TITAN study found that in patients with mCSPC treated with APA + ADT, survival, rPFS, time to PSA progression, and time to castration resistance were all statistically significantly improved in patients with evidence of a rapid and deep PSA response (either PSA90 or undetectable PSA at 3 months) compared to patients who did not have a PSA response^16^.

ADT and CAB continue to be used widely in Japan despite availability of new life-prolonging therapies. Use of ADT alone in other countries has declined over time but it remains commonly used, particularly in older patients and those with comorbidities^24^. In our study, patients started on CAB/ADT were somewhat older than patients started on other treatments but resembled the other treatment groups in terms of other characteristics, although a statistical comparison was not performed.

AAP was approved for use in Japan only for high-risk patients according to the LATITUDE study, which might explain why castration resistance-free survival in the AAP + ADT group was the lowest observed amongst ARPIs, although statistically significantly higher than CAB/ADT.

Strengths of this study are the large sample size and use of real-world data to build on clinical trial findings. Potential limitations are related to the MDV database; the database only captures in-hospital deaths which means that survival may be overestimated. Clinical information such as disease volume, Gleason score, and measures of function such as the ECOG performance score are not available in the database and could not be taken into account in our analysis. PSA analyses were restricted to the subgroup of approximately 15% patients with available PSA results in the MDV dataset and the follow-up up time was limited. The baseline PSA level was low, likely reflecting prior ADT treatment. Nevertheless, further marked reductions in PSA were observed after addition of an ARPI. Recently approved treatments such as darolutamide triple therapy were not reimbursed for mCSPC at the time of the study and could not be included. Additionally, reasons contributing to the choice of starting treatment were not available. Finally, patient background characteristics could not be fully adjusted in the analysis as information was lacking in the database for some patients, which could limit interpretation of the data.

## Conclusion

In conclusion, real-world evidence from a nationally representative claims database in Japan demonstrated that the use of APA + ADT as starting treatment for patients with mCSPC was associated with statistically significantly prolonged castration sensitivity and survival and induced faster and deeper PSA responses compared to traditional CAB or ADT. The data suggested clinically relevant benefits for patients with mCSPC started on APA + ADT.

## Electronic supplementary material

Below is the link to the electronic supplementary material.

Supplementary Material 1

## Data Availability

The data underlying this article were provided to Janssen Pharmaceuticals Kabushiki Kaisha by Medical Data Vision under license. Data will be shared on reasonable request to the corresponding author with permission of Medical Data Vision.
