# Supplementary material for: The impact of androgen receptor pathway inhibitors as starting treatment in metastatic castration-sensitive prostate cancer on patient outcomes (OASIS Japan)
Source: Sci Rep. 2025 Apr 19;15:13598. doi: 10.1038/s41598-025-93136-9 (PMC12009293; doi:10.1038/s41598-025-93136-9)
Supplement: Supplementary file 1 — Supplementary Material 1 [file 41598_2025_93136_MOESM1_ESM.docx]

**The impact of androgen receptor pathway inhibitors as starting treatment in metastatic castration-sensitive prostate cancer on patient outcomes (OASIS Japan)**

**SUPPLEMENT**

**Figure S1** Overall survival and time to undetectable PSA in patients with metastatic castration-sensitive prostate cancer, by starting treatment (CAB and ADT alone presented separately) – Kaplan-Meier method


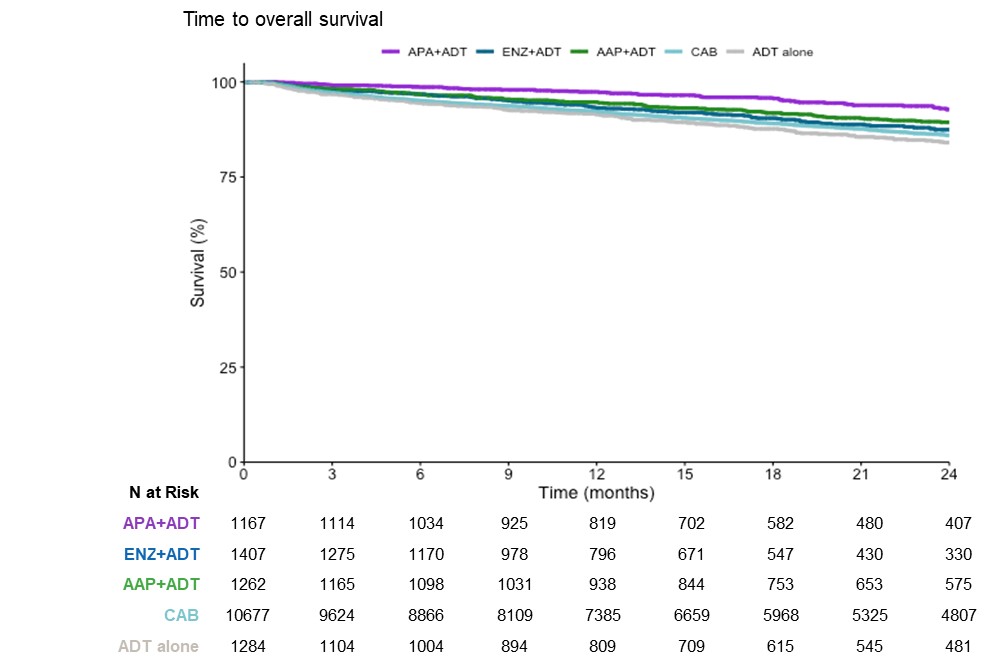


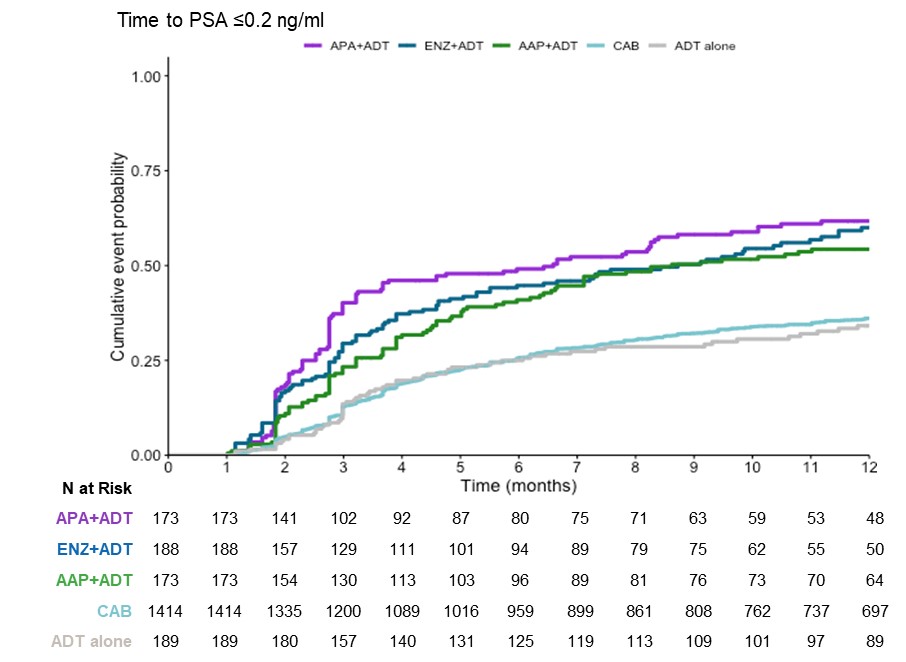


AAP, abiraterone acetate plus prednisone; ADT, androgen deprivation therapy; APA, apalutamide; CAB, combined androgen blockade; ENZ, enzalutamide; OS, overall survival
